# A genetic risk score of alleles related to MGUS interacts with socioeconomic position in a population-based cohort

**DOI:** 10.1038/s41598-022-08294-x

**Published:** 2022-03-15

**Authors:** Lisa Baak, Mirjam Frank, Jan Dürig, Ulrich Dührsen, Per Hoffmann, Markus M. Nöthen, Nico Dragano, Raimund Erbel, Karl-Heinz Jöckel, Börge Schmidt

**Affiliations:** 1 Institute for Medical Informatics, Biometry and Epidemiology, University Hospital Essen, University of Duisburg-Essen, Hufelandstr. 55, 45122 Essen, Germany; 2 Department of Haematology, University Hospital Essen, University of Duisburg-Essen, Essen, Germany; 3 Institute of Human Genetics, School of Medicine and University Hospital Bonn, University of Bonn, Bonn, Germany; 4 Institute of Medical Sociology, Medical Faculty, Centre for Health and Society, University of Düsseldorf, Düsseldorf, Germany

**Keywords:** Epidemiology, Risk factors

## Abstract

Environmental, genetic, and social factors are suggested to jointly influence monoclonal gammopathy of undetermined significance (MGUS), a precursor of multiple myeloma. Aim of this study was to investigate interactions between MGUS-related genetic variants and socioeconomic position (SEP) indicators education and income on MGUS in a population-based study. Two different MGUS-related genetic risk allele sum scores (GRS) were calculated based on recent genome-wide meta-analyses. Odds Ratios (OR) were estimated in 4329 participants including 238 MGUS cases to assess associations and multiplicative interaction. The relative excess risk due to interaction (RERI) was calculated to assess additive interaction. Both GRSs were associated with MGUS. A multiplicative interaction between one GRS and education was observed with genetic effects of OR 1.34 (95% CI 1.11–1.62) per risk allele in the highest and OR 1.06 (95% CI 0.86–1.31) in the lowest education group. A RERI of 0.10 (95% CI 0.05–0.14) also indicated additive interaction. Further, additive GRS by income interaction (RERI 0.07; 95% CI 0.01–0.13) for the same GRS was also indicated. Results indicate interaction between MGUS-related genetic risk and SEP. Non-genetic MGUS risk factors more common in higher education groups may influence the expression of MGUS-related genetic variants.

## Introduction

Monoclonal gammopathy of undetermined significance (MGUS) is an asymptomatic premalignant plasma-cell dyscrasia that is characterised by the presence of a monoclonal immunoglobulin (M-Protein). The prevalence of MGUS increases from age 50 onwards and its clinical relevance lies in the inherent risk of progression to hematologic malignancies such as multiple myeloma or other lymphoproliferative disorders as well as amyloidosis or light-chain deposition disease at an annual rate of ~ 1%^1,2^.

Case reports of familial clustering of multiple myeloma and case–control as well as cohort studies have provided strong evidence for increased risk of MGUS or multiple myeloma in first-degree relatives of affected patients^3–8^. In addition, several genome-wide association studies (GWASs) have identified multiple risk loci for MGUS and multiple myeloma. So far, common genetic variants at 23 loci have been associated with multiple myeloma risk^9–13^, which have also been shown to be at least weakly associated with MGUS^14–16^. However, genetic susceptibility of MGUS has only been studied to a limited extent, with two interrelated GWASs each identifying 10 different risk loci with suggestive evidence of genome-wide association^15,17^. Like other polygenetic risk factors for complex disorders, MGUS-related risk alleles only show small to moderate individual effects on the respective outcome. One explanation for this are possible gene-environment interactions.

Although preventable risk factors for MGUS are still largely unknown, inequalities in socioeconomic position (SEP) are discussed alongside other factors such as obesity, diabetes, smoking, diet, and other lifestyle factors as well as pesticide exposure and occupational factors^18–23^. However, most studies on modifiable risk factors have shown inconclusive results. So far, only one population-based study have indicated a positive association between SEP and MGUS while considering other discussed risk factors potentially mediating this effect^23^. As SEP can serve as a context defining variable that describes overall differences in risk-associated environments and health behaviours, it is hypothesized that SEP may affect disease via its influence on the social distribution of specific risk factors, which in turn have an impact on gene expression (i.e., gene-environment interaction)^24,25^.

The aim of this study was (1) to replicate the cumulative effect of genetic risk allele sum scores (GRSs) predisposing to MGUS using an independent sample of MGUS cases and (2) to investigate possible interactions between the GRSs and indicators of SEP (i.e., education and income) in a population based study cohort, where positive associations between indicators of SEP and MGUS have already been shown^23^. We calculated two separate MGUS GRSs each comprising 10 different risk loci from previous GWASs published in 2017^17^ and 2019^15^. To explore whether any detected GRS by SEP interactions are mediated by underlying interactions with other suggested MGUS risk factors, risk factors that were available for analysis in the study population (i.e., obesity, diabetes, smoking, dietary factors such as low fish, vegetable, and fruit consumption) were included in the analysis. As MGUS is one of the most common premalignant disorders in the general population, gaining a better understanding of the underlying causes and their interaction is important for the identification of factors suitable for disease prevention.

## Methods

### Study population

The present study is based on data of the prospective population-based Heinz Nixdorf Recall Study. The design and rationale of the study has been described in detail elsewhere^26^. Briefly, 4814 participants aged 45–75 years were recruited from 2000 to 2003 from three adjacent cities (Bochum, Essen, Mülheim/Ruhr) in an urban region in the western part of Germany. The first follow-up examination took place after a median time of ~ 5.1 years between 2005 and 2008, the second follow-up took place ~ 10.3 years after baseline between 2010 and 2015. The baseline response proportion of invited random sample of the population was 55.8%^27^. The study was approved by the ethics committee of the University of Duisburg-Essen and comprises extended quality management procedures including a certification according to DIN ISO 9001:2000. Written informed consent was obtained from all participants.

### Monoclonal gammopathy of undetermined significance (MGUS)

Serum samples were collected at baseline and prospectively at the 5-year and the 10-year follow-up examination at each visit and stored at − 80 °C. MGUS was assessed using standard serum electrophoresis combined with parallel screening immunofixation electrophoresis (scIFE) using pentavalent antisera (Hydragel 12 IF, Penta-Kit, Sebia, Fulda, Germany). In samples showing a visible or suspected monoclonal band, confirmatory IFE was performed using antisera against γ, α, μ, κ, and λ immunoglobulin chains. Results were assessed by a trained physician and MGUS cases were defined according to the International Myeloma Working Group criteria (i.e., including information on the detectable monoclonal protein on SPE and/or IFE, monoclonal protein concentration, laboratory results, and disease history)^28^.

Free kappa (κ) and free lambda (λ) immunoglobulin light chains (FLC) were determined using a Dade Behring BNII automated nephelometer (Siemens, Germany) utilizing a commercially available kit (FREELITE, The Binding Site Ltd, Birmingham, UK). Published ranges for κ and λ FLC were used as reference (3.3–19.4 mg/L and 5.7–26.3 mg/L, respectively)^29^. A pathological κ/λ FLC ratio was defined as < 0.26 or > 1.65 for participants with an estimated glomerular filtration rate (eGFR) of > 30 mL/min and as < 0.37 or > 3.1 for participants with an eGFR of < 30 mL/min^30^.

### Genetic data

Lymphocyte DNA was extracted from EDTA venous blood samples using the Chemagic Magnetic Separation Module I (Chemagen, Baesweiler, Germany) and genotyped using different Illumina microarrays according to the manufacturer’s protocols. Data from different Illumina genome-wide chips were imputed and then combined (Omni1-Quad n = 779, Omni1S n = 1348, HumanCoreExome n = 1747, Illumina OmniExpressv1.0 n = 457) resulting in a population of n = 4331 participants with an imputation quality of > 0.97 for all selected SNPs. Prior to imputation, quality control was applied separately for each chip on subject level including sex-, ethnicity- and relatedness-checks, excluding subjects with missing genotype data > 5%. Further, SNPs with a minor allele frequency (MAF) < 1%, a missing genotype frequency > 5% or a deviation from Hardy–Weinberg Equilibrium (HWE) (*p* < 10^−5^) were excluded. Imputation was carried out using IMPUTE v.2.3.1^31^ with reference data from 1000 Phase 3, release October 2014.

Two unweighted genetic risk allele sum scores for MGUS were constructed based on previous genome-wide association studies (GWAS) published in 2017^17^ (GRS_MGUS2017_) and 2019^15^ (GRS_MGUS2019_). Each GWAS have identified 10 loci associated with MGUS at a *p* value threshold of < 10^–5^. Of the GRS_MGUS2017_ risk loci, one SNP (rs10744861) was not available for 3874 participants. Therefore, we used rs1816225 as a proxy marker for those participants, which was in linkage disequilibrium of r^2^ = 0.86, based on European populations (CEU, TSI, FIN, GBR and IBS). Likewise, rs74998556 was used as a proxy marker with r^2^ = 0.73 for one SNP (rs74998556) of the GRS_MGUS2019_ score that was not available for 457 participants. The GRSs were then calculated by summing the total number of risk alleles for each individual across the selected SNPs.

### Indicators of SEP

Information on education and income was assessed by standardized face-to-face interviews at baseline examination. Education was defined as total years of formal education by combining school and vocational training according to the International Standard Classification of Education (ISCED-97)^32^. Education was included in all SEP stratified analyses categorized into four groups with the lowest educational group of ≤ 10 years (equivalent to a basic school degree with no vocational training) and the highest educational group of ≥ 18 years of education. For all regression models including SEP indicators education was dichotomized into low education (< 14 years) vs. high education (≥ 14 years). Income was defined as the monthly household equivalent income calculated by dividing the total household net income by a weighting factor for each household member^33^. Income was included in all SEP stratified analyses as a categorized variable using sex-specific quartiles. Income was dichotomized using sex specific medians for all regression models including SEP indicators. To take into account different mechanisms causing health inequalities, both SEP indicators were analysed separately^34,35^.

### Potential risk factors

Data on potential risk factors for MGUS were collected at study baseline (i.e., smoking, obesity, diabetes, low dietary intake of fish, vegetables, and fruits). Information on smoking was dichotomised into one group comprising current and past smoking (smoking cigarettes during the past year or having a history of smoking before the past year), and the second group of never smoking. Obesity was defined according to WHO criteria for individuals having a body mass index of 30 or more, utilizing standardized measurements of height and weight (kg/m^2^). Diabetes mellitus was defined as either of the following criteria: nonfasting glucose levels of 200 mg/dL or greater, fasting blood glucose levels of greater than 125 mg/dL, a reported history of diabetes mellitus, or intake of glucose lowering drugs. Dietary intake was assessed by a validated food frequency questionnaire (FFQ)^36^. Frequency of consumption was assessed using a five-point scale with the categories daily, 4–6 times/week, 1–3 times/week, 1–3 times/month, and hardly ever/never for each food item. Based on the FFQ, information for boiled vegetable, raw vegetable, and fruit consumption was dichotomized into low consumption (1–3 times/week or less) vs. high consumption (at least 4–6 times/week). Fish consumption was dichotomized into low consumption (1–3 times/month) vs. high consumption (at least 1–3 times/week)^37^.

### Statistical analyses

For the main analyses, 4329 participants with non-missing information on MGUS and genetic variants were included (Figure S1). Additional missing information on education (n = 12) and income (n = 267), smoking status (n = 6), obesity (n = 21), and dietary factors (n = 65–71) led to the exclusion of these participants from the respective analyses. Participants with missing information on income or education did not differ in rates of MGUS or in the GRS distribution compared to the analysis population. All analyses included both, prevalent MGUS cases at baseline and incident MGUS cases at the 5- and 10-year follow-up examination (i.e., having MGUS at least at one of the three examination dates).

First, age- and sex-adjusted logistic regression models were fitted to calculate odds ratios (OR) and their corresponding 95% confidence intervals (95% CI) to detect associations of the two SEP indicators and the two GRSs with MGUS in four separate regression models. In addition, the age- and sex-adjusted effect of the two GRSs on MGUS was also calculated stratified by SEP groups.

Second, to assess GRSxSEP interactions on the multiplicative scale, the GRS and SEP main effects as well as GRSxSEP interaction terms were included separately for the SEP indicators education and income as dichotomized variables in the regression models (base models). The regression coefficient of the interaction term in the logistic regression models calculated here reflect interaction on a multiplicative scale; however, it has been proposed that interaction described as departure from additivity of effects on disease events is better suited for indicating biological interaction^38^. Hence, the relative excess risk due to interaction (RERI) and the corresponding 95% CI was calculated to estimate interaction on the additive scale utilizing the regression coefficients of the logistic regression models including interaction terms.

Third, single reference joint effects of the GRS and SEP indicators were assessed by grouping GRS tertiles and SEP categories in all possible combinations into dummy variables that were then entered in regression models with the group of lowest GRS and lowest SEP as reference.

Finally, to investigate whether suspected GRSxSEP interactions were mediated by underlying interactions involving other potential MGUS risk factors, the base model was extended by GRS by risk factor and SEP by risk factor interaction terms and respective main effects in separate models for each risk factor. Single SNP analyses were performed accordingly assuming an additive genetic model. All analyses were performed using the statistical computing software R v3.5.3^39^ and Plink v1.07 for single SNP analyses for Windows^40^.

## Results

Characteristics of the study population are presented in Table 1. The total number of MGUS cases was 238 (5.5%) with women having a lower risk of MGUS than men. Clinical characteristics of MGUS cases are presented in Table S1. Mean values and standard deviations of MGUS-GRSs were 5.3 ± 2.0 (GRS_MGUS2017_) and 9.0 ± 1.9 (GRS_MGUS2019_), respectively. Both GRSs showed slightly higher average numbers of risk alleles for participants diagnosed with MGUS compared to non-MGUS participants.

**Table 1:** Characteristics of the study population stratified by MGUS status.

	All	MGUS	no MGUS
*n*	4329 (100%)	238 (5.5%)	4091 (94.5%)
Age (years)*	59.6 (± 7.8)	61.9 (± 7.7)	59.5 (± 7.8)
Sex (female)^†^	2165 (50.0%)	94 (39.5%)	2071 (50.6%)
GRS_MGUS2017_*	5.3 (± 2.0)	5.6 (± 2.1)	5.3 (± 2.0)
GRS_MGUS2019_*	9.0 (± 1.9)	9.3 (± 2.0)	8.9 (± 1.9)
Education (years of training)^†^ [n_miss_ = 12]			
< = 10 years	486 (11.3%)	20 (8.4%)	466 (11.4%)
11–13 years	2409 (55.8%)	119 (50.2%)	2290 (56.1%)
14–17 years	972 (22.5%)	66 (27.8%)	906 (22.2%)
> = 18 years	450 (10.4%)	32 (13.5%)	418 (10.2%)
Income (€/month)^‡^ [n_miss_ = 267]	1449 (1108–1875)	1619 (1150–2033)	1149 (1108–1875)
Current or past smoking^†^ [n_miss_ = 6]	2517 (58.2%)	149 (62.6%)	2368 (60.0%)
Obesity^†^ (BMI ≥ 30) [n_miss_ = 21]	1177 (29.1%)	74 (31.2%)	1103 (27.1%)
Diabetes mellitus^†^	588 (13.6%)	43 (18.1%)	545 (13.3%)
Dietary factors			
Low fish consumption^†^ [n_miss_ = 69]	2694 (63.2%)	148 (63.0%)	2546 (63.3%)
Low boiled vegetables consumption^†^ [n_miss_ = 67]	2695 (63.2%)	157 (66.8%)	2538 (63.0%)
Low raw vegetables consumption^†^ [n_miss_ = 71]	2937 (69.0%)	169 (72.2%)	2768 (68.8%)
Low fruit consumption^†^ [n_miss_ = 65]	1316 (30.8%)	74 (31.5%)	1242 (30.8%)

*Mean (± sd), ^†^Number(%), ^‡^Median (quartile range).

As expected, single SNP associations showed no genome-wide significance; however, magnitude and direction of effects were consistent to those reported in the original GWAS for 9/10 of the GRS_MGUS2017_-SNPs and 7/10 of the GRS_MGUS2019_-SNPs (Tables S2–S3). Both GRSs were associated with MGUS risk (GRS_MGUS2017_: OR, 1.08; 95% CI, 1.01–1.15; and GRS_MGUS2019_: OR, 1.12; 95% CI 1.04–1.20 per additional risk allele; Table 2). Both SEP indicators were also associated with MGUS in separate logistic regression models (Table 2). With the low education group (< 14 years of training) as reference, an OR of 1.43 (95% CI 1.07–1.90) was observed for the high education group (≥ 14 years of training). Similarly, the high income group (income > sex-specific median) showed an OR of 1.38 (95% CI 1.05–1.82) (Table 2).

**Table 2:** Sex-and age adjusted odds ratios (OR) and 95% confidence intervals (95% CI) for the main effects on MGUS status in four separate logistic regression models including either the MGUS-associated genetic risk scores (GRS), dichotomized education or income (lower category as reference).

Model	n (n_case_)	OR (95%CI)	*P*
GRS_MGUS2017_	4329 (238)	1.08 (1.01; 1.15)	0.02
GRS_MGUS2019_	4329 (238)	1.12 (1.04; 1.20)	1.44*10^–3^
Education (≥ 14 years)	4317 (237)	1.43 (1.07;1.90)	0.02
Income (high)	4062 (222)	1.38 (1.05; 1.82)	0.02

**Figure 1:**
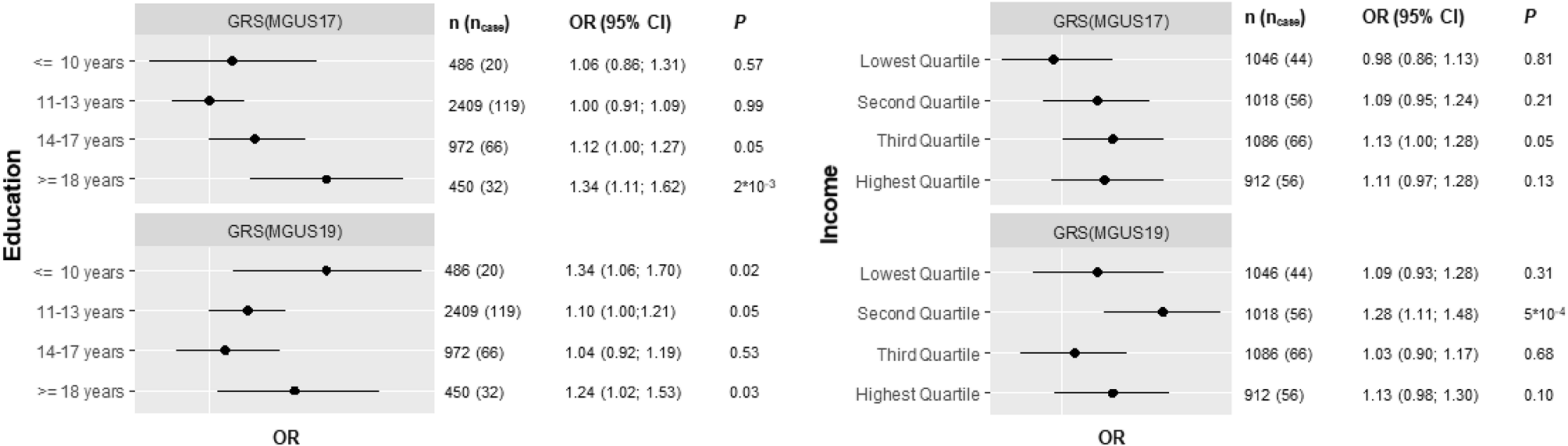
Sex-and age-adjusted odds ratios (OR) and 95% confidence intervals (95% CI) for the effect of genetic risk scores GRS_MGUS2017_ or GRS_MGUS2019_ on MGUS status, stratified by education groups (years) and income quartiles in logistic regression models.

Stratified analyses for the genetic effect on MGUS indicated stronger effects of the GRS_MGUS2017_ in higher SEP groups (Fig. 1), with the strongest effect in the highest education group (OR 1.34; 95% CI 1.11–1.62 per additional risk allele). A similar, although less pronounced trend of GRS_MGUS2017_ effects was observed for income quartiles with higher associations in Q2-Q4 compared to the lowest quartile. Associations of the GRS_MGUS2019_ were also stronger in the highest education group compared to the lower education groups; however, a strong effect of GRS_MGUS2019_ was also observed in the lowest education group. No clear trend was observed for the GRS_MGUS2019_ effect stratified by income (Fig. 1).

In analyses modelling the interaction between the GRSs and SEP, indication for a GRS_MGUS2017_ by education interaction was observed on the multiplicative scale showing an OR_interaction_ of 1.17 (95% CI 1.03–1.33) per additional risk allele for high (≥ 14 years) compared to low (< 14 years) education (Table 3). The RERI reflecting additive GRS_MGUS2017_ by education interaction effect on MGUS was 0.10 (95% CI 0.05–0.14) for high (≥ 14 years) vs. low (< 14 years) education (Table 3). Further, there was also indication for additive GRS_MGUS2017_ by income interaction on MGUS (RERI: 0.07; 95% CI 0.01–0.13 for high compared to low income). No indication for interaction was observed for using GRS_MGUS2019_ in the analyses (Table 3).

**Table 3:** Sex-and age-adjusted odds ratios (OR) and 95% confidence intervals (95% CI) for the effects on MGUS status in logistic regression models including main effects and interaction terms of the genetic effect (GRS_MGUS_) and dichotomised education or income as indicators of socioeconomic position (SEP) with the lower SEP category as reference group (relative excess risk due to interaction, RERI).

	MGUS ~ Education + GRS_MGUS2017_ + age + sex + education x GRS_MGUS2017_ *(n* = *4317; n*_*case*_ = *237)*		MGUS ~ Education + GRS_MGUS2019_ + age + sex + education x GRS_MGUS2019_ *(n* = *4317; n*_*case*_ = *237)*		MGUS ~ Income + GRS_MGUS2017_ + age + sex + income x GRS_MGUS2017_ *(n* = *4062; n*_*case*_ = *222)*		MGUS ~ Income + GRS_MGUS2019_ + age + sex + income x GRS_MGUS2019_ *(n* = *4062; n*_*case*_ = *222)*	
	OR (95% CI)	*P*	OR (95% CI)	*P*	OR (95% CI)	*P*	OR (95% CI)	*P*
Age	1.04 (1.02; 1.06)	2.49*10^–06^	1.04 (1.03; 1.06)	1.98*10^–06^	1.04 (1.02; 1.06)	4.22*10^–06^	1.04 (1.03; 1.06)	2.73*10^–06^
Sex (male)	1.39 (1.05; 1.86)	0.02	1.41 (1.07; 1.89)	0.02	1.60 (1.21; 2.12)	9.90*10^–3^	1.62 (1.23; 2.16)	6.88*10^–4^
GRS_MGUS2017_	1.01 (0.93; 1.10)	0.81			1.04 (0.94; 1.14)	0.44		
GRS_MGUS2019_			1.14 (1.04; 1.24)	5*10^–3^			1.19 (1.07;1.32)	1.34*10^–3^
Education (≥ 14 years)	0.58 (0.26; 1.27)	0.17	1.95 (0.52; 7.23)	0.32				
Education x GRS_MGUS2017_	1.17 (1.03; 1.33)	0.02						
Education x GRS_MGUS2019_			0.97 (0.84; 1.11)	0.64				
Income (> median)					0.89 (0.40; 1.95)	0.76	3.62 (0.94; 14.09)	0.06
Income x GRS_MGUS2017_					1.08 (0.95; 1.24)	0.24		
Income x GRS_MGUS2019_							0.90 (0.78; 1.04)	0.16
RERI (95% CI)	0.10 (0.05; 0.14)		0.06 (− 0.03; 0.14)		0.07 (0.01; 0.13)		0.07 (− 0.14; 0.28)	

The analysis of single reference joint effects of GRS_MGUS2017_ and SEP indicators on MGUS showed an increasing trend of effect size estimates with increasing years of education in groups with the highest GRS_MGUS2017_ tertile (Table 4). Compared to the reference group with the lowest education and lowest GRS_MGUS2017_ tertile, participants within the highest GRS_MGUS2017_ tertile and highest education category showed on average a fivefold higher MGUS risk (OR 4.99; 95% CI 1.87–15.77). Single reference joint effect analyses of income and GRS_MGUS2017_ also indicated the strongest MGUS risk for participants within the highest income quartile and highest GRS_MGUS2017_ tertile compared to the reference group (Table 4).

**Table 4:** Sex-and age adjusted odds ratios (OR) and 95% confidence intervals (95% CI) for the single reference joint effects of the genetic effect (GRS_MGUS2017_) and SEP indicators on MGUS status calculated separately for income and education with the group of being in the low genetic risk score tertile and the lowest SEP category as reference.

	n (n_case_)	OR	95%CI	*P*
**Education**				
**Education ≤ 10 years**				
GRS_MGUS2017_ lower tertile	160 (5)	Ref	…	…
GRS_MGUS2017_ middle tertile	188 (10)	1.77	0.62; 5.81	0.31
GRS_MGUS2017_ highest tertile	138 (5)	1.22	0.33; 4.47	0.76
**Education 11–13 years**				
GRS_MGUS2017_ lower tertile	791 (45)	2.07	0.88; 6.07	0.13
GRS_MGUS2017_ middle tertile	870 (39)	1.62	0.68; 4.77	0.32
GRS_MGUS2017_ highest tertile	748 (35)	1.68	0.70; 4.97	0.29
**Education 14–17 years**				
GRS_MGUS2017_ lower tertile	347 (20)	1.94	0.75; 6.01	0.20
GRS_MGUS2017_ middle tertile	326 (18)	1.88	0.72; 5.86	0.23
GRS_MGUS2017_ highest tertile	299 (28)	3.20	1.29; 9.71	0.02
**Education ≥ 18 years**				
GRS_MGUS2017_ lower tertile	147 (7)	1.84	0.57; 6.45	0.31
GRS_MGUS2017_ middle tertile	160 (8)	1.93	0.62; 6.61	0.26
GRS_MGUS2017_ highest tertile	143 (17)	4.99	1.87; 15.77	2.59*10^–3^
**Income**				
**Income Q1**				
GRS_MGUS2017_ lower tertile	338 (15)	Ref	…	…
GRS_MGUS2017_ middle tertile	360 (21)	1.34	0.71; 2.80	0.34
GRS_MGUS2017_ highest tertile	348 (8)	0.51	0.20;1.20	0.13
**Income Q2**				
GRS_MGUS2017_ lower tertile	358 (18)	1.24	0.61; 2.53	0.56
GRS_MGUS2017_ middle tertile	359 (16)	1.07	0.52; 2.23	0.85
GRS_MGUS2017_ highest tertile	301 (22)	1.85	0.95; 3.72	0.07
**Income Q3**				
GRS_MGUS2017_ lower tertile	357 (18)	1.29	0.64; 2.64	0.48
GRS_MGUS2017_ middle tertile	404 (19)	1.21	0.60; 2.46	0.60
GRS_MGUS2017_ highest tertile	325 (29)	1.29	1.25; 4.59	0.01
**Income Q4**				
GRS_MGUS2017_ lower tertile	315 (21)	1.77	0.89; 3.57	0.10
GRS_MGUS2017_ middle tertile	318 (14)	1.14	0.54; 2.42	0.74
GRS_MGUS2017_ highest tertile	279 (21)	2.01	1.02; 2.07	0.05

The addition of interaction terms with other potential MGUS-risk factors (i.e., smoking, obesity, diabetes, low dietary intake of fish, vegetables, and fruits) to the logistic regression model including the GRS_MGUS2017_xEducation interaction term did not substantially change the effect size estimate of the GRS_MGUS2017_xEducation interaction, neither on the multiplicative nor on the additive scale (Table 5).

**Table 5:** Changes in the interaction effect of the genetic effect (GRS_MGUS2017_) by dichotomised education (E) on MGUS (base model) in separate sex-and age-adjusted logistic regression models additionally including the main effects and respective interaction terms for each potential risk factor (odds ratios, OR; 95% confidence intervals, 95% CI; relative excess risk due to interaction, RERI).

MGUS risk factor	n (n_case_)	OR (95% CI) _(GRSxE)_	*P*_(GRSxE)_	RERI (95% CI)
Base model	4317 (237)	1.17 (1.03; 1.34)	0.02	0.10 (0.05; 0.14)
Current or past smoking	4317 (237)	1.19 (1.04; 1.36)	0.01	0.07 (− 0.01; 0.16)
Obesity	4298 (236)	1.17 (1.03;1.34)	0.02	0.09 (0.04; 0.15)
Diabetes mellitus	4317 (237)	1.18 (1.03; 1.34)	0.01	0.11 (0.05; 0.16)
Low fish consumption	4252 (234)	1.18 (1.03; 1.34)	0.02	0.10 (0.03;0.17)
Low boiled vegetables consumption	4254 (234)	1.17 (1.03; 1.34)	0.02	0.07 (− 0.01; 0.15)
Low raw vegetables consumption	4250 (233)	1.19 (1.04; 1.36)	0.01	0.14 (0.06;0.22)
Low fruit consumption	4256 (234)	1.17 (1.03; 1.34)	0.02	0.10 (0.04; 0.15)

In the single SNP interaction analysis using education as SEP indicator, 7 of the 10 SNPs included in the GRS_MGUS2017_ were directionally consistent to the overall GRS_MGUS2017_ by education interaction effect on the multiplicative scale (Table S4), while this was true for 8 of the 10 SNPs regarding interaction on the additive scale. SNP rs3118053 presented the strongest indication for interaction with education on both scales.

## Discussion

The aim of this study was (1) the replication of cumulative genetic risk factors predisposing to MGUS and (2) to investigate possible interactions between genetic risk allele sum scores and indicators of SEP and their impact on MGUS in a population-based study cohort employing MGUS cases not included in previous GWAS. Associations of two different sum scores of MGUS-related genetic variants with MGUS were observed. In addition, results gave indication for positive interaction between one MGUS-related GRS (GRS_MGUS2017_) and SEP indicator education on both the additive and multiplicative scale and for income on the additive scale, resulting in stronger associations between MGUS-related genetic risk and MGUS in higher SEP groups. Effect size estimates for the GRS_MGUS2017_ by education interaction remained unchanged after including discussed MGUS risk factors (i.e., smoking, obesity, diabetes, low dietary intake of fish, vegetables, and fruits) into regression analysis, suggesting that these factors do not explain the observed GRS_MGUS2017_ by education interaction. Further, results of stratified analyses support the suspected interaction with the strongest genetic effect on MGUS observed in the highest education group. In addition, joint effects of all possible combinations of GRS_MGUS2017_ tertiles and education groups showed the strongest effect on MGUS for participants with highest genetic risk within the highest education group.

In the present study, the cumulative effect of MGUS-related genetic risk alleles reported in two different GWAS by Thomsen et al.^15,17^ was replicated utilising cumulative GRSs. The associations between SNPs effect alleles and MGUS in both previous studies have been reported to reach suggestive evidence of genome-wide association (*p* < 10^–5^), probably due to small numbers of MGUS cases. The first GWAS from 2017 comprised 243 MGUS cases and 1285 controls from Germany with a replication of three SNPs in an independent Czech cohort of 294 cases and 272 controls. The second GWAS from 2019 comprised in total 992 MGUS cases and 2900 controls based on three different data sets including the previously mentioned German cohort, a Czech cohort of 288 cases and 600 controls and additionally a Swedish cohort including 461 cases and 1,025 controls. Since none of the 10 loci associated with MGUS risk identified in the first GWAS were replicated in the second (probably due to fluctuations of allele frequencies in smaller GWAS samples), we decided a priori to investigate associations of the reported SNP sets separately in our study population. Another recent GWAS including 754 MGUS cases from Mayo Clinic and MD Anderson could only replicate one of the reported MGUS risk loci^16^. This lack of replication may be explained by underlying population differences and differences in the distribution MGUS risk types between study populations. However, both MGUS-related genetic sum scores showed an association with MGUS in our study population with GRS_MGUS2019_ showing a slightly stronger association with MGUS than the GRS_MGUS2017_. In contrast to the present study, the underlying genetic variants of the GRSs calculated here were identified in GWAS in which MGUS cases were mainly not selected from MGUS screening studies, but from clinical MGUS collectives.

The main finding of this study is the indication for stronger associations between GRS_MGUS2017_ and MGUS in groups of higher SEP. Results of stratified analyses revealed the strongest genetic effect of GRS_MGUS2017_ on MGUS in the highest education group and joint effects of all possible combinations of GRS_MGUS2017_ tertiles and education groups showed the strongest effect on MGUS for participants with highest genetic risk within the highest education group. These findings together with the effect size estimates for the GRS_MGUS2017_ by education interaction term and the RERI estimate gave strong indication for positive interaction that is more than the product and the sum of both independent variables, meaning that the presence of these underlying genetic variants is accompanied with a higher risk for developing MGUS in higher educational groups. Results for interaction between SEP indicator income and GRS_MGUS2017_ were less pronounced; however, we still observed an indication for additive interaction between GRS_MGUS2017_ and income and stronger associations of GRS_MGUS2017_ with MGUS in higher income groups. SEP stratified analyses of GRS_MGUS2019_ on MGUS showed more heterogeneous results across the strata. However, it showed also stronger associations in the highest education group compared to all other groups.

As SEP usually has no direct effect on disease, but influences disease development indirectly via risk factors that are unequally distributed across SEP groups^23^, results of the present analysis indicate that MGUS risk factors more prevalent in higher SEP groups may also impact the expression of MGUS-related genetic effects. However, since the inclusion of potential MGUS risk factors that were available for analysis did not explain the observed GRS_MGUS2017_ by education interaction, other risk factors for MGUS not included in the analysis may be underlying the observed interaction effect. In previous studies, stronger genetic effects on health risks in higher SEP groups have also been explained by a lack of competing non-genetic risk factors in higher SEP groups^41^. However, in the present study both, a higher overall MGUS risk as well as stronger MGUS-related genetic effects were observed in higher SEP groups. The stronger overall MGUS risk in higher SEP groups suggests some sort of MGUS risk factor that is more prevalent in higher SEP groups and that may also affect MGUS risk via its interaction with MGUS-related genetic factors.

Besides its population-based study design, strengths of this study include the assessment of both prevalent and incident MGUS cases over a 10-year follow-up period using a sensitive diagnostic screening approach, the inclusion of two different SEP indicators as well as the inclusion of other potential MGUS risk factors into analyses. Moreover, interaction analyses were not merely based on testing GRSxSEP interaction terms and additional RERI calculation, but also on stratified analyses and analyses of joint effects. A limitation of the study is the lack of urine protein measurements as well as imaging and bone marrow biopsy results for giving more detailed information on MGUS diagnosis and its severity. Thus, a small fraction of MGUS cases and rare plasma cell dyscrasias might have been missed. Moreover, due to the sample size the statistical power for single SNP analyses was limited. Furthermore, complex interdependencies of the analysed risk factors cannot be ruled out for contributing to the observed GRSxSEP interaction. However, with regard to potential model overfitting the sample size of the study was not suitable for simultaneously exploring potential interactions between all factors included in the analysis.

In conclusion, results of the present study provide further evidence for associations of previously reported genetic variants for MGUS risk and indicate stronger associations between one MGUS-related GRS (GRS_MGUS2017_) and MGUS in higher SEP groups in a population-based study sample. This suggests that the genetic risk related to MGUS may not act independently from potentially preventable risk factors. However, the present study was not able to identify potential MGUS risk factors underlying the observed GRS by SEP interaction, indicating the existence of unknown risk factors for MGUS not included in the analysis that are more common in people with higher SEP that seem to influence the expression of MGUS-associated genetic variants. However, further studies are needed to investigate MGUS risk factors including genetic variants and SEP effects on MGUS to reliably identify more distinct subgroups with higher exposure-specific disease risk and to gain further insights into the biology of MGUS.

## Supplementary Information

Supplementary Information.

## Data Availability

Due to data security reasons (i.e., data contain potentially participant identifying information), the Heinz Nixdorf Recall Study does not allow sharing data as a public use file. However, others can access the data used upon request, which is the same way the authors of the present paper obtained the data. Data requests can be addressed to: recall@uk-essen.de.
