# Supplementary material for: A genetic risk score of alleles related to MGUS interacts with socioeconomic position in a population-based cohort
Source: Sci Rep. 2022 Mar 15;12:4409. doi: 10.1038/s41598-022-08294-x (PMC8924237; doi:10.1038/s41598-022-08294-x)
Supplement: Supplementary file 1 — Supplementary Information. [file 41598_2022_8294_MOESM1_ESM.pdf]

Supplement

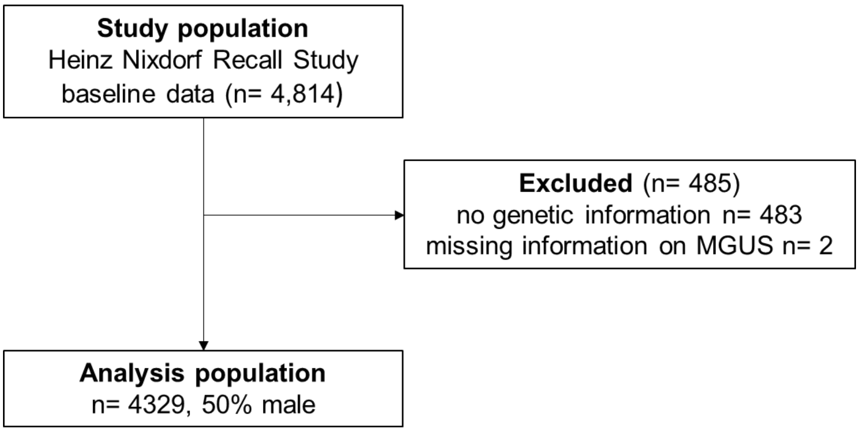

Figure S1. Flowchart describing the number of participants of the Heinz Nixdorf Recall study cohort included in the analysis.

Table S1. Characteristics of study participants with MGUS at the time of MGUS detection stratified by sex.

	All	Women	Men
**n***	238 (100%)	94 (39.5%)	144 (60.5%)
**Age at MGUS**†	65.05 (±8.37)	65.23 (±8.96)	64.92 (±7.99)
**M-protein Isotype*** [n_{miss}=5]			
IgG	142 (60.9%)	58 (62.4%)	84 (60.0%)
IgA	34 (14.6%)	14 (15.1%)	20 (14.3%)
IgM	47 (20.2%)	17 (18.3)	30 (21.4%)
other	10 (4.3%)	4 (4.3%)	6 (4.3%)
**Incident MGUS over 10 years*** [n_{miss}=1]			
Incident MGUS	85 (35.9%)	41 (43.6%)	44 (30.8%)
Prevalent MGUS	152 (64.1%)	53 (56.4%)	99 (69.2%)

*Number(%), †Mean (±sd)

Table S2. Sex- and age-adjusted odds ratios (OR) and 95% confidence intervals (95% CI) for the effect of each $GRS_{MGUS2017}$ –associated single-nucleotide polymorphism (SNP) on MGUS status.

Chr.	Position	SNP	Risk Allele	Other Allele	RAF	OR	95% CI	*P*
1	58225485	rs3131740	C	G	0.43	1.06	0.88-1.28	0.51
1	58226522	rs3118053	T	C	0.49	1.07	0.89-1.29	0.49
6	164043825	rs6933936	T	C	0.27	1.12	0.91-1.37	0.28
7	7972285	rs10251201	C	T	0.49	1.06	0.88-1.28	0.53
7	87164836-7	rs28381958	A	G	0.16	1.18	0.92-1.50	0.18
8	2646618	rs974120	C	T	0.09	1.11	0.79-1.52	0.52
12	116122376	rs10744861*	T	C	0.12	1.29	0.99-1.67	0.06
13	74500970	rs9318227	T	C	0.12	1.02	0.76-1.35	0.88
18	33205897	rs16966921	G	A	0.07	0.94	0.62-1.35	0.74
19	45602781	rs10405859	T	C	0.38	1.16	0.96-1.40	0.11

*for 3874 participants proxy SNP (rs1816225) with R^2 =0.86

Table S3. Sex- and age-adjusted odds ratios (OR) and 95% confidence intervals (95% CI) for the effect of each $GRS_{MGUS2019}$ –associated single-nucleotide polymorphism (SNP) on MGUS status.

Chr.	Position	SNP	Risk Allele	Other Allele	RAF	OR	95% CI	*P*
1	214301323	rs3009934	T	G	0.69	0.89	0.72; 1.10	0.27
3	41753647	rs9848754	T	C	0.16	1.12	0.87; 1.43	0.36
3	104051156	rs73180532	C	A	0.47	1.13	0.94; 1.37	0.19
4	108802381	rs72888948	A	C	0.10	1.03	0.74; 1.39	0.87
8	105068489	rs9656789	G	A	0.81	0.97	0.78; 1.23	0.80
8	143466597	rs4928692	A	G	0.81	1.48	1.15; 1.95	0.003
10	7250346	rs7920332	C	G	0.56	1.24	1.02; 1.51	0.04
14	69108086	rs12436964	T	A	0.34	1.25	1.03; 1.51	0.02
15	64535700	rs4561409	T	C	0.31	1.18	0.97; 1.44	0.10
17	16839782	rs74998556*	T	A	0.16	0.94	0.70; 1.23	0.65

*****for 457 participants proxy SNP (rs74998556) with R^2 =0.73

Table S4. Sex- and age-adjusted odds ratios (OR) and 95% confidence intervals (95% CI) for the main effect and the interaction of each $GRS_{MGUS2017}$ –associated single-nucleotide polymorphism (SNP) with education categories (reference: low) on MGUS (relative excess risk due to interaction, RERI).

SNP	Risk Allele		OR (95% CI)	*P*	RERI (95% CI)
rs3131740	C	SNP	0.95 (0.74; 1.21)	0.68	
		Age	1.04 (1.03; 1.06)	$2*10^{-6}$	
		Sex	1.40 (1.05; 1.86)	0.02	
		Education	1.13 (0.74; 1.71)	0.58	
		SNPxEducation	1.34 (0.92; 1.95)	0.12	0.36 (-0.03; 0.74)
rs3118053	T	SNP	0.85 (0.66; 1.08)	0.18	
		Age	1.04 (1.03; 1.06)	$2*10^{-6}$	
		Sex	1.40 (1.05; 1.86)	0.02	
		Education	0.77 (0.56; 1.26)	0.30	
		SNPxEducation	1.81 (1.23; 2.66)	0.003	0.56 (0.30; 0.82)
rs6933936	T	SNP	1.13 (0.86; 1.47)	0.38	
		Age	1.04 (1.03; 1.06)	$2*10^{-6}$	
		Sex	1.40 (1.05; 1.86)	0.02	
		Education	1.45 (0.99; 2.11)	0.05	
		SNPxEducation	0.97 (0.63; 1.48)	0.90	0.14 (-0.55; 0.58)
rs10251201	C	SNP	1.09 (0.85; 1.39)	0.49	
		Age	1.04 (1.03; 1.06)	$2*10^{-6}$	
		Sex	1.40 (1.05; 1.86)	0.02	
		Education	1.54 (0.94; 1.50)	0.08	
		SNPxEducation	0.93 (0.63; 1.36)	0.71	-0.07 (-0.59; 0.45)
rs28381958	A	SNP	1.09 (0.77; 1.49)	0.62	
		Age	1.04 (1.03; 1.06)	$2*10^{-6}$	
		Sex	1.40 (1.05; 1.86)	0.02	
		Education	1.34 (0.95; 1.87)	0.09	
		SNPxEducation	1.20 (0.72; 1.98)	0.47	0.32 (-0.37; 1.00)
rs974120	C	SNP	0.93 (0.96; 1.80)	0.74	
		Age	1.04 (1.03; 1.06)	$1*10^{-6}$	
		Sex	1.40 (1.05; 1.86)	0.02	
		Education	1.31 (0.96; 1.80)	0.09	
		SNPxEducation	1.52 (0.79; 2.92)	0.21	0.61 (-0.31; 1.52)
rs10744861	T	SNP	1.37 (0.96; 1.90)	0.07	
		Age	1.04 (1.03; 1.06)	$2*10^{-6}$	
		Sex	1.40 (1.05; 1.86)	0.02	
		Education	1.50 (1.08; 2.08)	0.02	
		SNPxEducation	0.84 (0.48; 1.42)	0.52	-0.15 (-0.97; 0.67)
rs9318227	T	SNP	0.79 (0.52; 1.17)	0.27	
		Age	1.04 (1.03; 1.06)	$2*10^{-6}$	
		Sex	1.40 (1.05; 1.86)	0.02	
		Education	1.24 (0.90; 1.71)	0.02	
		SNPxEducation	1.72 (0.97; 3.07)	0.06	0.66 (-0.04; 1.36)
rs16966921	G	SNP	0.91 (0.53; 1.48)	0.70	
		Age	1.04 (1.03; 1.06)	$2*10^{-6}$	
		Sex	1.40 (1.05; 1.86)	0.02	
		Education	1.40 (1.04; 1.90)	0.03	
		SNPxEducation	1.13 (0.51; 2.44)	0.77	0.12 (-0.84; 1.09)
rs10405859	T	SNP	1.14 (0.89; 1.45)	0.31	
		Age	1.04 (1.03; 1.06)	$1*10^{-6}$	
		Sex	1.40 (1.05; 1.86)	0.02	
		Education	1.37 (0.89; 2.10)	0.15	
		SNPxEducation	1.04 (0.71; 1.52)	0.85	0.12 (-0.37; 0.59)
